# CFRP Thin-Ply Fibre Metal Laminates: Influences of Ply Thickness and Metal Layers on Open Hole Tension and Compression Properties

**DOI:** 10.3390/ma13040910

**Published:** 2020-02-18

**Authors:** Benedikt Kötter, Julian Karsten, Johann Körbelin, Bodo Fiedler

**Affiliations:** Institute of Polymer and Composites, Hamburg University of Technology, Denickestraße 15, 21073 Hamburg, Germanyfiedler@tuhh.de (B.F.)

**Keywords:** stainless steel foil, stress distribution, hybrid material, non-destructive testing, digital image correlation

## Abstract

Thin-ply laminates exhibit a higher degree of freedom in design and altered failure behaviour, and therefore, an increased strength for unnotched laminates in comparison to thick-ply laminates. For notched laminates, the static strength is strongly decreased; this is caused by a lack of stress relaxation through damage, which leads to a higher stress concentration and premature, brittle failure. To overcome this behaviour and to use the advantage of thin-ply laminates in areas with high stress concentrations, we have investigated thin-ply hybrid laminates with different metal volume fractions. Open hole tensile (OHT) and open hole compression (OHC) tests were performed with quasi-isotropic carbon fibre reinforced plastic (CFRP) specimens. In the area of stress concentration, 90° layers were locally substituted by stainless steel layers of differing volume fractions, from 12.5% to 25%. The strain field on the specimen surface was evaluated in-situ using a digital image correlation (DIC) system. The embedding of stainless steel foils in thin-ply samples increases the OHT strength up to 60.44% compared to unmodified thin-ply laminates. The density specific OHT strength is increased by 33%. Thick-ply specimens achieve an OHC strength increase up to 45.7%, which corresponds to an increase in density specific strength of 32.4%.

## 1. Introduction

Fibre reinforced composites (FRPs) are used in structural applications, such as aircraft construction, automotive manufacturing, shipbuilding and sports equipment because of their excellent weight-specific mechanical properties. Fastener-based joining techniques such as bolting or riveting are commonly used in these applications, as parts become highly maintainable and can be easily disassembled and reattached. However, for FRPs such as carbon fibre reinforced plastics (CFRPs), riveting is not a material-appropriate design, due to their low bearing strength and high notch sensitivity [1,2]. Therefore, different attempts to reduce the notch sensitivity of composites are utilised; e.g., local thickening of the laminate [3], optimised laminate layup and stacking sequence [4,5], local inserts [6], z-pinning [4,7] and hybridisation with other materials [8,9,10].

High-performance carbon-fibre reinforced plastics (CFRPs) are widely applied as structural materials in applications where a low density combined with high stiffness and strength is required. Due to the multi-scale nature and the different constituents, the failure in composites is complex. Matrix-cracks, delamination and fibre failure can occur, and failure at the micro-level influences the failure process at all higher levels. As a result, not only do the mechanic properties of the constituents of the composite define the strength and failure process, but the lay-up design and the layer thickness do as well [11,12,13,14,15]. Thin-ply laminates are characterised by a layer thickness of less than <60 µm. These layer thicknesses became available through the advancement and industrialisation of the spread-tow process as presented by Kawabe [16] and Sihn [13]. By reducing the thickness of the single layer, the number of layers can be chosen to be more load-dependent. This increases the degrees of freedom in the orientation and the quantity of the individual layers.

Thin layers suppress transverse microcracking and free edge delamination. As a result, the occurring failure modes change from complex multi-mode failure to a quasi-brittle failure, from thick- to thin-ply [13,14,15,17,18]. For unnotched quasi-isotropic laminates, this leads to a significant increase in tensile strength, which utilises the potential of the constituents [13,19]. The damage initiation changes with decreasing layer thickness to higher strains. Thin-ply specimens show little to no visible premature damage before ultimate failure. Under compressive loading of quasi-isotropic specimens, a similar behaviour is observed. The compressive strength increases with decreasing layer thickness. In addition to the changing failure behaviour, the material quality also plays an important role. Due to the small layer thicknesses and the spreading process, a more homogeneous fibre distribution and smaller resin-rich regions are achieved [15].

For notched laminates, the static strength is strongly decreased, because the lack of stress relaxation through damage leads to a higher stress concentration and premature, brittle failure compared to thick-ply laminates [13,15,18], which is limiting to the design space of thin-ply composites.

One possibility to change the failure mechanisms and improve the mechanical properties of notched specimens is to insert metallic layers into the laminate. Fibre metal laminates (FMLs) show improved load-bearing and a progressive failure mechanism. Fibre metal laminates are utilised to combine the favourable properties of metallic and composite behaviours. The main advantages compared to pure fibre reinforced composites are: quasi ductile failure behaviour due to the additional plasticity of the steel foils [3,20], better energy absorption under tensile loads, better structural integrity in crash tests and local electrical conductivity, which allows amongst other things for non-destructive testing (NDT) and structural health monitoring (SHM) [21].

With conventional layer thicknesses, the adhesion between metal and matrix is a major challenge. Interlaminar shear forces, caused by thermal loads from the curing process and external loads, act between composite layers and metal foils. The strength of the interface is therefore of high importance, as it influences the failure process of the laminate extensively. Due to the higher number of layers and the associated higher number of interfaces, the shear stresses are lower and the pretreatment process of the metal has less influence. In addition, a high number of layers offers freedom of design concerning hybrid composites. Various proportions of steel or positions of the steel in the laminate can be realised. An additional advantage is the use of thin and more flexible stainless steel foils. Especially in components with complex geometries and curved areas like the wings of an aircraft, adapting and shape forming of the material is necessary. Thicker steel foils would be needed to be preformed before lamination, whereas thin steel foils can be shaped during the process of laminating up to a level of deformation similar to the CFRP layers. A first study concerning the combination of thin-ply CFRP and stainless steel foils was published in 2015 by Masani et al. [22]. They investigated open hole tensile (OHT) and load-bearing properties of thin-ply fibre metal laminates with a CFRP-layer and metal foil thickness of 30 µm and a steel volume content of 25%. An increase of up to two times in bearing strength was encountered. The specific bearing strength, bearing strength in relation to the density of the specimen, is lower than that of CFRP without stainless steel foils. However, according to Studer et al. [20], it is sufficient to use local stainless steel reinforcements in regions of load introduction or high local stresses; as a result the component density would decrease. The aim of this study is to analyse a new method to improve the open hole tensile and compressive strength of thin-ply laminates by replacing 90°-CFRP-layers with stainless steel foils as patches with the same layer thickness.

## 2. Materials and Methods

### 2.1. Materials and Specimen Preparation

In this study, austenitic steel alloy inserts 1.4310 (X10CrNi18-8) from Knight Strip Metals Ltd. (Hertfordshire, UK) were used as metal reinforcement foil in CFRP. The alloy has a metastable austenitic structure due to its high chromium and nickel content, which strengthens the material during processing due to work hardening in a cold rolling process. The tensile strength is between 500 and 700 MPa, with a yield strength of 210 MPa and a Young’s modulus of 200 GPa. Due to its good mechanical and durability properties, the alloy is used in aircraft construction and automotive engineering, and has been used in multiple previous studies on fibre metal laminates [8,23,24,25,26,27].

**Figure 1:**
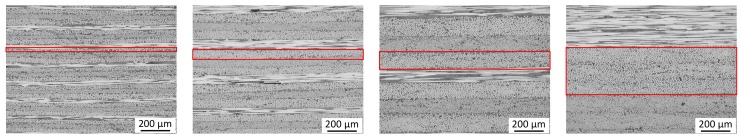
Microsections of the used CFRP laminates, left to right: 30, 60, 120 and 240 gsm (block-scaling 2 × 120 gsm).

The steel foils were cut with a precision cutter for electronic boards, which results in no visible deformation at the edges of the foils. The samples for the tension tests of the stainless steel foils have the dimensions 250 mm × 25 mm. The nominal thicknesses of the foils are 0.03 mm and 0.15 mm. Unidirectional CFRP prepregs with fibre areal weights (FAWs) of 30 gsm, 60 gsm and 120 gsm are used. Other FAWs (150 gsm, 240 gsm) are achieved via block-scaling. The prepreg was manufactured by North Thin Ply Technology Switzerland (NTPT), using T700S carbon fibres from Toray Carbon Fibres America Inc (CMA) and ThinPreg 402 epoxy resin from NTPT. The experimentally determined mechanical properties of the prepreg system are shown in Table 1. Tensile tests were conducted in compliance to ASTM D 3039 [28] standard with a quasi-isotropic layup and specimen dimensions of 1.82 mm × 25 mm × 150 mm. Five specimens were tested per configuration. In Figure 1, microsections of the tensile specimens show the difference in thickness of the individual layers in red. In order to compare different steel foil surface pretreatments, interlaminar shear strength (ILSS) tests were carried out. For ILSS tests, an unidirectional prepreg system HexPly M21/35%/268/T800S from Hexcel Corporation, with M21 epoxy based resin and CMA’s T800S carbon fibres, was used. The single-layer thickness of this prepreg system is 0.262 mm.

**Figure 2:**
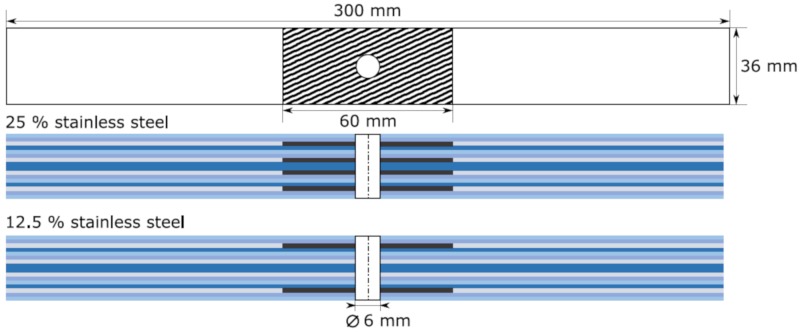
Schematic drawing of the sample design for OHT and OHC tests according to the standards ASTM D5766 [30] and ASTM D6484 [31].

The prepreg was cut using a computer numerical control (CNC) cutter Aristomat TL 1625 from ARISTO Graphic System GmbH and Co. KG. and laminated by hand. For every fourth prepreg layer, a pre-evacuation was performed to further compress the laminate and to prevent voids and air inclusions. The different laminate layups are shown in Table 2. Depending on the layer structure, some 90°-layers were replaced by stainless steel foils, which exhibit a limited contribution to the global load carrying capacity; 60 mm wide stainless steel foils were inserted as patches in the area of the centred hole of the specimens. This is shown schematically in Figure 2. The black areas represent the stainless steel foil. In the case of the hybrid laminates with a metal volume content of 12.5%, the stainless steel layers were placed on the outside, so that the benefit of the bending stiffness of the metal foils could be utilised under compressive load. For each ILSS sample, a steel foil with a layer thickness of 0.15 mm was placed in the middle of the laminate. The remaining layers are unidirectional in 0°-orientation. The Layup is listed in Table 2. The sample dimensions of the ILSS samples were 40 mm × 12 mm × 6 mm according to the ASTM D2344 standard [29].

The surfaces of stainless steel foils were pretreated to ensure sufficient adhesion between the stainless steel and the epoxy resin matrix. Six pretreatment methods which had been proven to be effective were investigated [32]. For all methods presented, the first step was to clean the stainless steel foils with acetone. Two chemical etching methods were chosen. For the first method, the samples were chemically etched by sulphuric acid (30% concentration at 60 °C for 4 min) followed by a solution of 22–28 parts by weight (PBW) of sulphuric acid and 2–3 PBW of potassium dichromate. For the second etching method, the samples were primarily preparated with hydrofluoric acid (4% concentration at 50 °C for 20 min), followed by a solution of 22–28 PBW of sulphuric acid and 2–3 PBW of potassium dichromate.

Plasma surface treatment was chosen for methods three and four. In one group, the stainless steel foils were plasma treated directly after the cleaning process with acetone, and the other group was sanded with 500 grit silicon carbide sandpaper before plasma treatment. The plasma system in use was a SmartPlasma 10 system by Plasma Technology GmbH (parameters: 300 W, 90 s, 0.3 mbar).

In addition, the sol-gel process was used. This surface pretreatment was prepared according to the procedure outlined by 3M Aerospace and Aircraft Maintenance Division with the 3M surface pretreatment AC-130-2. The surface pretreatment AC-130-2 is a water-based system and can be used in combination with different metals. According to 3M, the achieved benefits of the pretreatment are in the same range or better than conventional etching processes. An additional advantage of the system is the formation of a chemical bond between the metal and the matrix without using potentially carcinogenic and allergenic chromates. The sol-gel surface pretreatment can be easily applied to the metal by spraying, brushing or immersion, allowing for an on-site use on the aircraft. For one sample group, the surface was roughened with 500 grit silicon carbide sandpaper, which increases the metal bonding surface and removes coarse contamination. Further, the foil surface was cleaned with acetone to remove any sanding residue and grease from the surface. After this, the AC-130-2 was applied with an immersion bath at room temperature and dried for 60 min. This increases the adhesion effect due to an increased surface area and more chemical bonds between the metal surface and the epoxy resin. The second group was directly pretreated with AC-130-2 after cleaning the surface with acetone without any surface roughening. To compare the results with samples without stainless steel foil, some reference samples were prepared for the ILSS tests.

The laminates (420 mm × 300 mm) were cured in an autoclave at 120 °C and 4 bar in a nitrogen atmosphere. The cured plates were milled using an Isel Euromod 25 three axis milling machine. The specimen dimensions are determined according to the standards for OHT (ASTM D5766 [30]), OHC (ASTM D6484 [31]) and ILSS (ASTM D2344 [29]) tests. The dimensions are 300 mm × 36 mm × 3.84 mm with a central hole (diameter: 6 mm) for the OHT and OHC samples. After milling, the edges of the samples were polished and all samples dried in a vacuum furnace at 40 °C for 12 h before they are tested.

### 2.2. Experimental Methods

All mechanical tests were performed under constant ambient conditions (temperature 23 °C, relative humidity 50%). An universal testing machine Z2.5 by ZwickRoell GmbH and Co. KG (Ulm, Germany) was used for the tensile tests of the stainless steel foils. The foils were clamped using mechanical clamping jaws. The test speed was set to 3 mm/min. For the strain measurement, cross-head position and an optical camera measuring system from ZwickRoell were used. In order to achieve this, two high contrast markings were applied to the specimen surface and tracked by the camera system.

The ILSS tests were carried out according to ASTM D2344 [29] on a ZwickRoell Z10 universal testing machine. The support radius was 1.5 mm and the radius of the compression cylinder was 3 mm. The span length was chosen as proposed by the standard (24 mm) and the speed of testing applied was 1 mm/min. Displacement and strain measurement were recorded using the traverse path of the upper stamp, directly connected to the cross-head displacement.

Open hole tensile and compression tests were performed in accordance to ASTM D5766 [30] and ASTM D6484 [31] using a ZwickRoell Z400 universal testing machine. Mechanical wedge clamps were used for the tensile tests, whereby the forces were introduced into the specimen via shear forces. The cross-head speed was set to 2 mm/min. The displacement and strain were recorded using mechanical displacement transducers (MultiXtens from ZwickRoell), and the digital image correlation (DIC) system Aramis 4M system from GOM GmbH. A high contrast speckle pattern (consisting of white and black acrylic paint) sprayed onto the specimen surfaces allowed for computer-aided image evaluation and strain monitoring with the software GOM Correlate Professional. The camera focus was set directly at the open hole in the centre of the specimen.

For the open hole compression tests, a cross-head speed of 2 mm/min and an anti-buckling support as specified in the ASTM were used. The mechanical loads were applied via the end faces of the specimens. The displacement was determined over the cross-head traverse, since there was no possibility of using the MultiXtens due to the anti-buckling support. Furthermore, the DIC system was used, recording the sample through a small window inside the anti-buckling support.

For the micrographs, the tested specimens were embedded in epoxy resin so that the fracture surfaces were not damaged during further mechanical processing. Depending on the specimen and the fracture pattern, the specimens were sawn and embedded in resin so that they could be polished. The subsequent polishing was done in several steps. First, the samples were ground with sandpaper in various grit sizes and then polished with diamond suspension up to a particle size of 3 µm.

## 3. Results and Discussion

**Figure 3:**
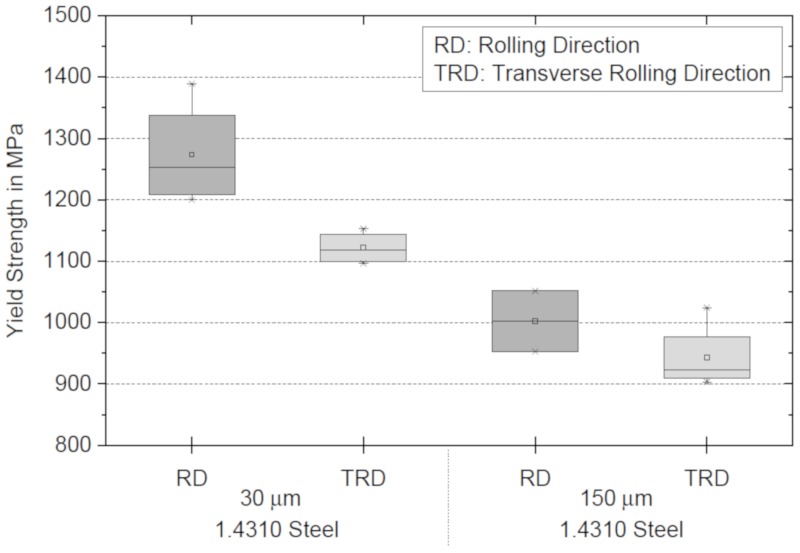
Yield strength regarding foil thickness and orientation during the rolling process.

Figure 3 shows the results of the stainless steel foil tensile tests. The yield strength is plotted over the foil layer thickness in relation to the rolling orientation of the stainless steel. RD (rolling direction) means that the main load direction is parallel to the rolling direction of the foil. Accordingly, TRD (transverse rolling direction) means that the main load direction is perpendicular to the rolling direction of the foil. A comparison of the measured yield strength reveals that a significant difference between the rolling and transverse rolling direction is apparent in the case of the thin foils. For the thick foils, no difference could be found. However, strong evidence of an increase of yield strength with decreasing foil thickness was found. The yield strength of the thin foil in the rolling direction is 27.1%, and transverse to the rolling direction it is 18.9% higher than for the thick foils. The results of the yield strength and the results of the fracture strength are shown in Table 3. No significant difference in fracture strength was found. This can be explained by the work or strain hardening of the metal foils.

**Figure 4:**
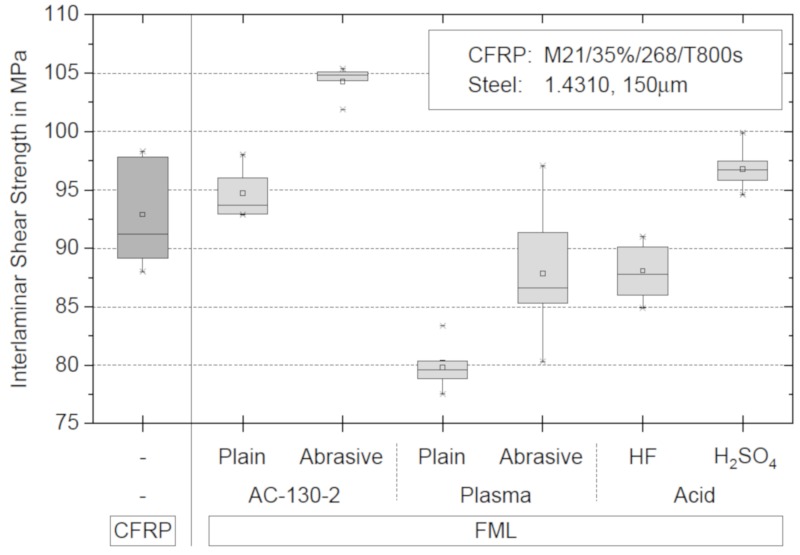
Interlaminar shear strengths (ILSSs) of different surface pretreatments of the stainless steel foils according to ASTM D2344 [29].

It is difficult to compare the results of the specimens with stainless steel with the specimens without stainless steel, because ILSS samples with stainless steel do not have a symmetrical structure and therefore cannot be regarded as ideal specimens. The lower and upper parts of the specimen have different bending stiffness. However, the different pretreatment methods can be compared, and significant differences between the pretreatments can be seen. A comparison reveals that the interlaminar shear strength of the specimen pretreated with abrasive paper and AC-130-2 is most pronounced. Figure 4 shows the results of the ILSS tests. CFRP without stainless steel foil reaches 92.91 MPa, which is 2.91 MPa above the value specified by the manufacturer Hexcel. It is also interesting to note that the interlaminar shear strength of the stainless steel decreases as a result of plasma treatment, which is contrary to the current literature. From the results, it follows that due to the high interlaminar shear strength and the low standard deviation, the sol-gel process with the combination between abrasive paper and AC-130-2 surface treatment system from 3M was used for further open hole tensile and compressive tests.

**Figure 5:**
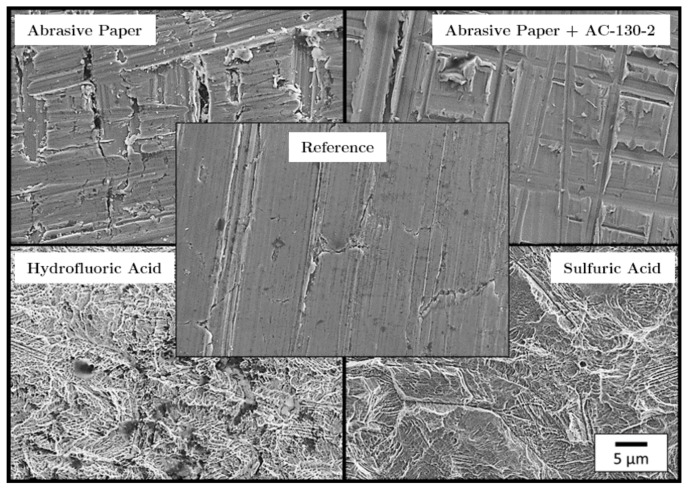
Scanning electron microscope images of the pretreated stainless steel surfaces.

Figure 5 shows scanning electron microscope (SEM) images of the surface of pretreated stainless steel foils. It is noticeable that the etched surfaces have a finely structured surface, which in turn indicates theoretically good adhesion. Since the the sol-gel process merely forms a chemical intermediate layer (film) on the stainless steel surface and thus does not cause any geometric changes to the surface, no difference can be detected between the surfaces of the samples ground and those ground and treated with AC-130-2 using SEM.

**Figure 6:**
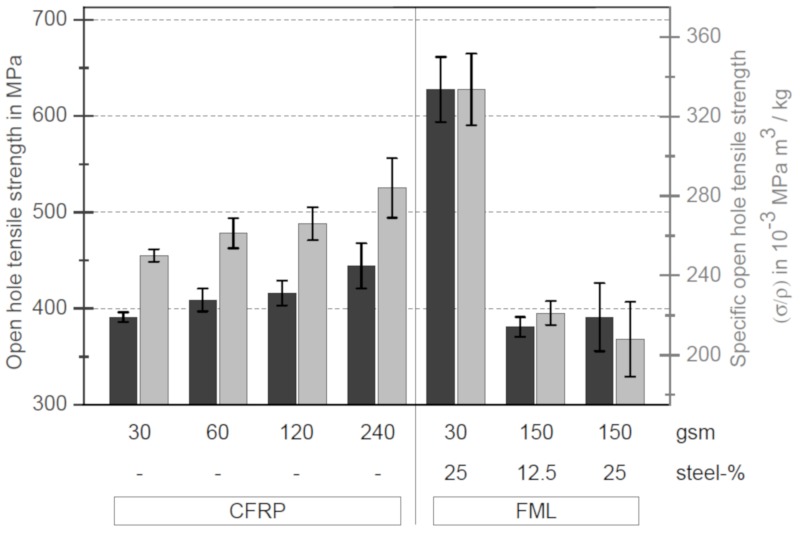
Open hole tensile strength and specific open hole tensile strength of CFRP samples with and without steel foils and different layer thicknesses.

Figure 6 illustrates the open hole tensile strengths (black, left axis) of the samples with and without stainless steel patches. The ordinate on the right side shows the specific open hole tensile strength (grey). The specific open hole tensile strength means that the open hole tensile strength of a sample is related to its density. The calculation of the density takes into account that the stainless steel foils are only used as patches, as they will be used in practical applications; see Figure 2.

**Figure 7:**
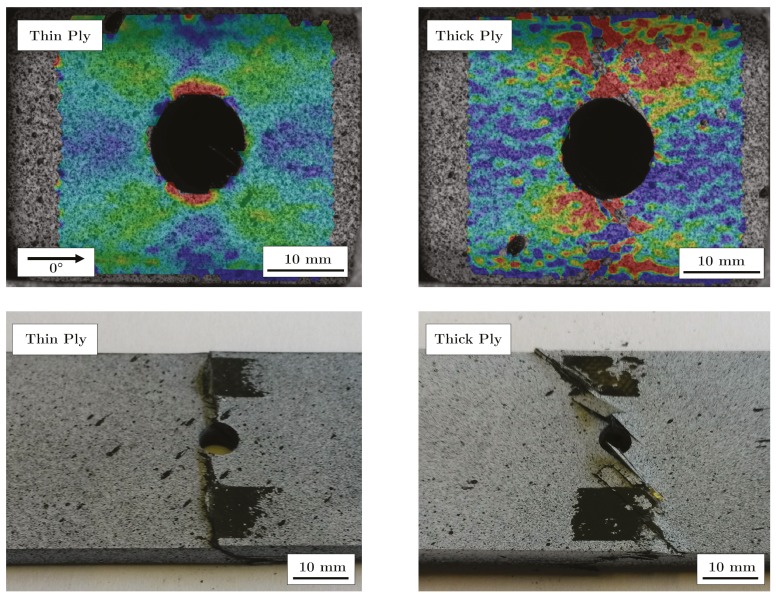
Digital image correlation (DIC) and fracture patterns of the open hole tensile specimens.

From Figure 6 it is obvious that the open hole tensile strength decreases significantly with decreasing layer thickness without stainless steel foil. The open hole tensile strength of the thin-ply specimens decreases by 12% compared to the samples with the thickest layer thickness. In contrast, the tensile strength of quasi-isotropic (QI) samples without a hole increases from 736.86 MPa (thick-ply) to 956.59 MPa (thin-ply), which corresponds to an increase of 29.8%. This can be explained by the changing fracture behaviour of the specimens, as described by Sihn [13] or Amacher [15]. In the case of thicker layers, the material is damaged at the hole during loading. Interfibre fractures and delaminations occur. The different damages at the surface could be detected with the DIC system. Figure 7 shows two fracture patterns on the left side after the tensile tests, where the left specimen is a thin-ply and the right specimen a thick-ply. The right side shows DIC images taken one second before final failure. The DIC images illustrate the strain field on the surface of the samples. The strain field can be used to draw qualitative conclusions about the stress field of the samples. In the case of thick-ply samples, predamage was detected before ultimate failure. As a result of this predamage, there is a relaxation process near the hole, and the stress peak near the hole will be reduced. Stresses are deflected by the damage in the material. In contrast, for thin-ply laminates, no predamage was visible until final failure with the DIC system. The samples failed in a brittle way, perpendicular to the load direction. Other studies used an acoustic emission system [13] and showed that there is no predamage before final failure within thin-ply laminates. The result of this behaviour is reflected in the fracture patterns (left side, Figure 7). It can be seen that in the case of the thin layer specimens, no delaminations are visible, which suggests that the critical failure mechanism must be fibre breakage. Only a partial detachment of the upper layer can be seen. In contrast, in the case of specimens with thicker layer delaminations and pull-outs, a mixed failure mode can be found.

**Figure 8:**
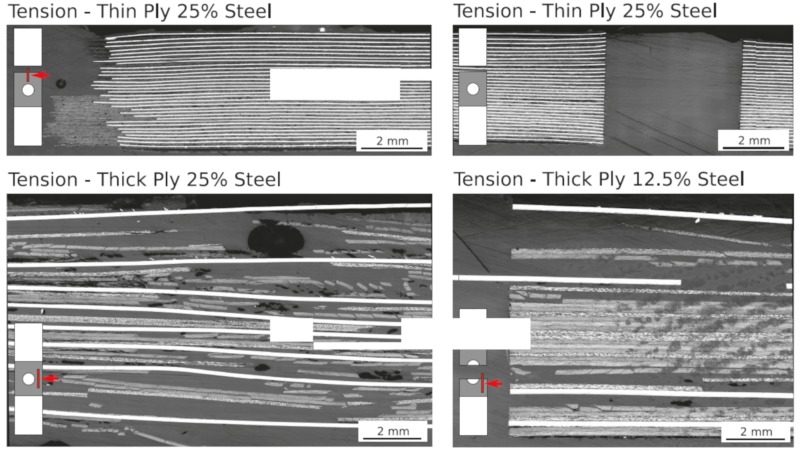
Microsections of the fracture surfaces of the open hole tension specimens.

For comparison purposes, Figure 6 shows on the right side the specimens with stainless steel. In the case of thin-ply specimens with a stainless steel content of 25%, the strength increases by 60.44%. Hybridisation with stainless steel foils locally increases the strength of the specimen and reduces the stress within the CFRP layers. Crack propagation at the hole is suppressed. The potential of the fibres can be further exploited. Most thin layer specimens with stainless steel failed at the transition zone between the area with and without stainless steel. This is also visible in the microsections in Figure 8. The upper left and right images show a thin-ply hybrid sample after final failure.

Due to the transition from stainless steel foils to 90°-CFRP-layers, stress concentrations occur, which could be increased by local defects. The microsections in Figure 8 show a small difference in the locations of the transitions between steel foils and matrix. The positions of the foils vary on average by 0.3 mm. In addition, some waviness of the foils or deformation at the edges can increase the stress concentration at the transition zone, and resin rich areas appear in the transition zone. Another disadvantage of this design is the local stiffness discontinuity due to the discontinuous transition between metal layers and 90° CFRP layers. Nevertheless, it should be mentioned that although high stresses were present, no delaminations are visible. This shows the advantage of thin-ply hybrid materials. Due to a large number of layers and the associated interfaces, the interlaminar shear strength between the layers is lower, so that the surface pretreatment selected here was sufficient.

**Figure 9:**
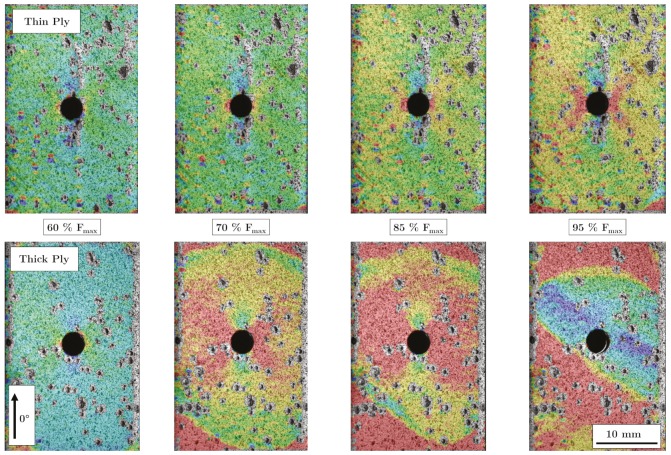
DIC open hole tensile samples at 60%, 70%, 85% and 95% of maximum force (top, thin-ply samples; bottom, thick-ply samples.

Figure 9 exhibits DIC images for selected loads. The upper four images show the damage process of a thin-ply sample. It can be seen that there are no large delaminations due to a shift in the upper layer. At the stress of 70% of the maximum stress, a stress peak is visible at the hole as well as stress peaks at the outer edges of the transition zone (red areas in the lower left and right corner of the image). However, these spread very slowly compared to the thick-ply specimens. The thick layer samples (lower images) show a delamination growth starting from the transition zone and the hole at 70% of the maximum stress. At 60% no delaminations are visible yet (left picture). As the load increases, the delaminations increase and move towards each other until the complete area of the sample in the area of the stainless steel shows delaminations.

In addition to the DIC images, the delaminations that occur can also be seen in the microsections in Figure 8. All delaminations are between the stainless steel foils and the matrix layers. The bonding between the stainless steel and the matrix was not sufficient. The interlaminar shear stress between steel and matrix was higher than the bonding strength between them.

However, for technical applications, the specific strength is more valuable, as it provides information on whether it is worthwhile to use such a material in the future. Even the strength of thin-ply samples in relation to the density increases by 33.14%; see Figure 6. This shows that by adding stainless steel foils as patches to make hybrid materials, an increase in strength relative to their densities can be achieved.

**Figure 10:**
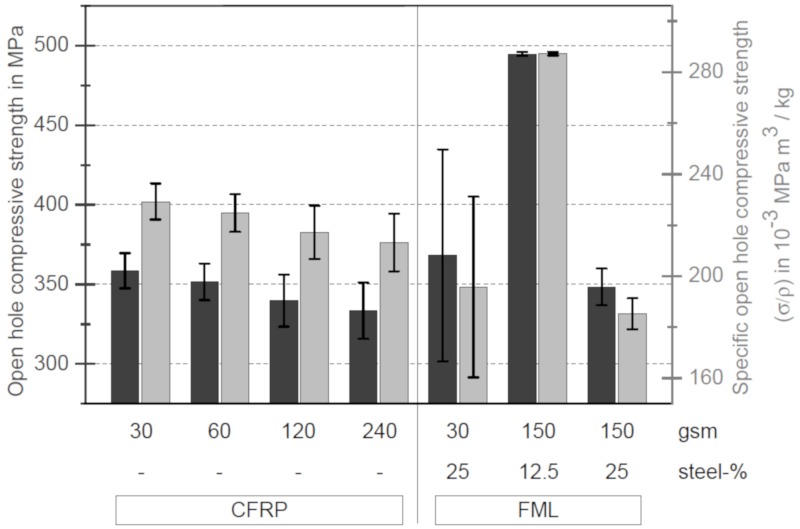
Open hole compressive strength and specific open hole compressive strength of CFRP samples with and without steel foils and different layer thicknesses.

**Figure 11:**
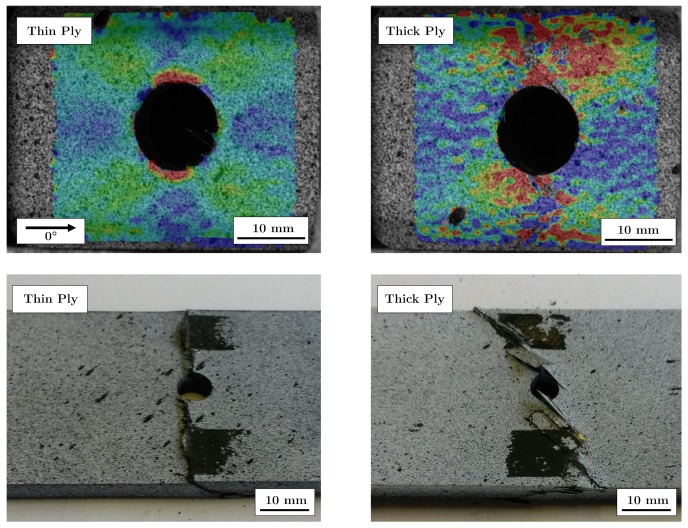
DIC (top) and fracture patterns (bottom) of the open hole compressive specimens without stainless steel.

The results of the open hole compressive tests are shown in Figure 10. The bar chart shows that there is no difference in open hole compressive strength between the specimens without stainless steel foils. However, strength is increasing with decreasing layer thickness. The strength of the thin-ply specimens is 7.5% higher than that of the thick-ply specimens. Similar results were obtained by Yokozeki et al. [33]. In his study, the strength of the thin-ply samples increased by 9%. The increase of the strength can be explained by the changing failure behaviour and the tension. In the case of thin layer specimens, the formation and spread of delaminations are suppressed. The critical failure occurred in the formation of a kink band through the whole thickness of the specimen. This can also be seen in Figure 11. On the left side, DIC images of a thin- (left DIC image) and a thick-ply (right DIC image) sample one second before failure are shown. In the case of thin-ply samples, no previous damage could be detected before final failure, whereas delaminations and fibre breaks of the surface of the thick-ply specimens were visible. The failure of the thick-ply samples is a combination of fibre kinking and delaminations. This combination results in final failure, as shown in Figure 11.

**Figure 12:**
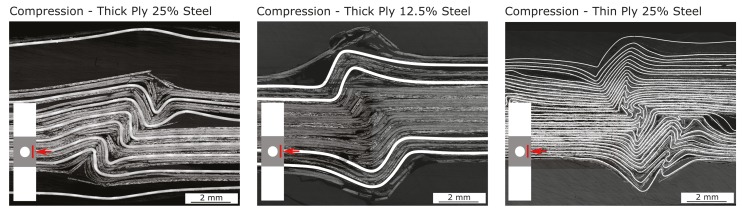
Microsections of the fracture surfaces of the open hole tension and compression specimens.

Concerning the hybrid samples, a significant increase in strength can be observed in the case of the thick-ply samples with a steel content of 12.5%. The open hole compressive strength increased from 340 MPa to nearly 500 MPa, an improvement of 47%. The other configurations did not show any major improvements. In the case of thick-ply samples with a steel content of 25%, the OHC strength did not change, and in the case of thin-ply hybrid specimens a large variation in the results could be observed. Some samples showed an improvement in OHC strength from 333.4 MPa to 436.5 MPa, and others a decrease to 303.3 MPa. The microsections (Figure 12) show the different failure behaviours.

In the case of thick-ply specimens with a steel content of 25%, delaminations occur between the stainless steel foil and the matrix, as is already the case under tensile load. Depending on which side the delaminations occur first, there is no symmetric bending stiffness, and the samples preferably kink to one side. This can also be seen in the fracture patterns or microsections. Due to this failure behaviour, no improvement in strength could be observed. In contrast, thick-ply specimens with a steel content of 12.5% have a 47% higher open hole compressive strength. The sample is supported by the increased bending stiffness of the hybrid composite due to the stainless steel layers and buckling is suppressed. No major delaminations can be detected within the sample such that a behaviour usual for composite materials can be seen here, whereby this is further strengthened by the outer steel layers as already mentioned.

The thin-ply hybrid specimens exhibit no predamage until final failure. No deformations in Z-directions (perpendicular to the sample surface) could be found via DIC. The microsection in Figure 12 displays numerous kinks in the specimen. Some kinks are local kink bands and other extend globally over several layers. The steel layers with a thickness of 30 µm have low compressive stiffness and due to small defects like waviness of the foil or voids lead to local deformations and kinks. The open hole compressive strength of the thin-ply hybrid specimens shows a large standard deviation based on this local deformations and bucklings. Specimens with a low content or number of local kinks exhibit a higher strength. Specimens with a high content or a high number of local kinks exhibit a lower strength.

As in the case of the tensile results, the specific open hole strength is shown in grey in Figure 10. The specific open hole compressive strength shows that in the case of thick-ply specimens with a steel content of 25%, there is a decrease in the specific strength. In the case of the thin-ply specimens with a steel content of 25%, a increase or decrease strength can be determined depending on the number of kinks. Only in the case of thick-ply specimens with a steel content of 12.5%, an improvement in the specific strength can be detected, which can be explained by the anti-buckling support of the laminate by the outer steel layers.

## 4. Conclusions

This study shows that the hybridisation of thin-ply CFRP samples with stainless steel foil patches increases the open hole tensile strength by up to 60.44% compared to CFRP samples. Even if the strength is normalised to the density of the samples, the OHT strength is increased by up to 33%. Hybridisation with stainless steel foils locally increases the strength of the specimen and reduces the stress within the CFRP layers. Crack propagation at the hole is suppressed. The potential of the fibres can be further exploited. For thick-ply hybrid samples, no improvement in OHT strength could be found. The laminates failed due to the formation of delaminations between the stainless steel foils and the matrix. The compression test showed different results. The thin-ply hybrid and the thick-ply hybrid samples with a steel content of 25% exhibit no improvement in open hole compressive strength. In the case of the specimens with thin layers, many kinks could be found which led to premature failure. The hybrid samples with the thicker layers and 25% steel failed due to delaminations. The thick-ply samples with a steel content of 12.5% exhibited an improvement in open hole compressive strength up to 47%. Due to the higher local bending stiffness of the hybrid material, buckling of the sample is suppressed. In summary, the hybridisation of CFRP laminates with stainless steel foils in exchange for the 90°-CFRP-layers can improve the OHT and OHC strength. Additionally, the specific OHT and OHC strength increase, so that this hybrid material could be an opportunity to reduce the notch sensitivity of composites, especially for thin-ply composites. For further investigations, the transition zone should be modified so that a strong local stiffness discontinuity can be avoided.

## Figures and Tables

**Table 1:** Ply thickness dependent tensile properties of quasi-isotropic CFRP-laminates (ASTM D 3039) [28].

FAW	Lay-up	Tensile Strength	Young’s Modulus
30 gsm	[45/90/−45/90]_8s_	956.59 ± 31.81 MPa	47.88 ± 1.65 GPa
60 gsm	[45/90/−45/90]_4s_	963.66 ± 18.21 MPa	50.02 ± 1.60 GPa
120 gsm	[45/90/−45/90]_2s_	825.49 ± 24.56 MPa	48.13 ± 2.54 GPa
240 gsm	[45_2_/90_2_/−45_2_/90_2_]_s_	736.86 ± 32.61 MPa	47.30 ± 1.47 GPa

**Table 2:** Laminate layups for the OHT, OHC and ILSS tests.

Test Setup	Configuration (SF: Steel Foil)	Layup	Number of Specimen
OHT/C	CFRP: 30 gsm	[45/−45/0/90]_16s_	5
OHT/C	CFRP: 30 gsm; SF: 25% (0.03 mm)	[45/−45/0/SF]_16s_	4
OHT/C	CFRP: 60 gsm	[45/−45/0/90]_8s_	5
OHT/C	CFRP: 120 gsm	[45/−45/0/90]_4s_	5
OHT/C	CFRP: 150 gsm; SF: 12.5% (0.15 mm)	[(45/−45/SF/0)_2_/(45/-45/0/90)_2_]_s_	3
OHT/C	CFRP: 150 gsm; SF: 25% (0.15 mm)	[45/−45/SF/0]_4s_	3
OHT/C	CFRP: 240 gsm	[45/−45/0/90]_2s_	5
ILSS	CFRP: 268 gsm	[0]_16_	5
ILSS	CFRP: 268 gsm; SF: 6.25% (0.15 mm)	[(0_7_/SF)/0_8_]	5

**Table 3:** Measured mechanical properties of stainless steel (1.4310) foils.

Foil Thickness in m	Orientation	Strength in MPa	Yield Strength in MPa
30	RD	1347.3±52.7	1273.8±85.8
30	TRD	1410.2±30.8	1121.7±27.0
150	RD	1337.8±71.1	1002.2±69.9
150	TRD	1371.3±8.3	943.4±55.0

## Abbreviations

The following abbreviations are used in this manuscript:

MDPI	Multidisciplinary Digital Publishing Institute
OHT	open hole tension
OHC	open hole compression
CFRP	carbon fibre reinforced plastic
gsm	grams by square meters
QI	quasi-isotropic
UD	Unidirectional
FML	fibre metal laminate
NDT	non-destructive testing
SHM	structural health monitoring
NTPT	north thin ply technology
CMA	Toray Carbon Fibres America, Inc
ILSS	interlaminar shear strength
CNC	computer numerical control
ASTM	American Society for Testing and Materials
SF	steel foil
SEM	scanning electron microscope
DIC	digital image correlation
